# Simplified dolutegravir dosing for children with HIV weighing 20 kg or more: pharmacokinetic and safety substudies of the multicentre, randomised ODYSSEY trial

**DOI:** 10.1016/S2352-3018(20)30189-2

**Published:** 2020-08-04

**Authors:** Pauline D J Bollen, Cecilia L Moore, Hilda A Mujuru, Shafic Makumbi, Adeodata R Kekitiinwa, Elisabeth Kaudha, Anna Parker, Godfrey Musoro, Annet Nanduudu, Abbas Lugemwa, Pauline Amuge, James G Hakim, Pablo Rojo, Carlo Giaquinto, Angela Colbers, Diana M Gibb, Deborah Ford, Anna Turkova, David M Burger, ODYSSEY trial team

**Affiliations:** a Department of Pharmacy, Radboud Institute for Health Sciences, Radboud university medical center, Nijmegen, Netherlands; b Medical Research Council Clinical Trials Unit, University College London, London, UK; c University of Zimbabwe Clinical Research Centre, Harare, Zimbabwe; d Joint Clinical Research Centre, Mbarara, Uganda; e Joint Clinical Research Centre, Kampala, Uganda; e Baylor College of Medicine Children's Foundation, Kampala, Uganda; f Hospital 12 de Octubre, Madrid, Spain; f University of Padova, Padova, Italy

## Abstract

**Background:**

Paediatric dolutegravir doses approved by stringent regulatory authorities (SRAs) for children weighing 20 kg to less than 40 kg until recently required 25 mg and 10 mg film-coated tablets. These tablets are not readily available in low-resource settings where the burden of HIV is highest. We did nested pharmacokinetic substudies in patients enrolled in the ODYSSEY-trial to evaluate simplified dosing in children with HIV.

**Methods:**

We did pharmacokinetic and safety substudies within the open-label, multicentre, randomised ODYSSEY trial (NCT02259127) of children with HIV starting treatment in four research centres in Uganda and Zimbabwe. Eligible children were randomised to dolutegravir in ODYSSEY and weighed 20 kg to less than 40 kg. In children weighing 20 kg to less than 25 kg, we assessed dolutegravir's pharmacokinetics in children given once daily 25 mg film-coated tablets (approved by the SRAs at the time of the study) in part one of the study, and 50 mg film-coated tablets (adult dose) or 30 mg dispersible tablets in part two of the study. In children weighing 25 kg to less than 40 kg, we also assessed dolutegravir pharmacokinetics within-subject on film-coated tablet doses of 25 mg or 35 mg once daily, which were approved by the SRAs for the children's weight band; then switched to 50 mg film-coated tablets once daily. Steady-state 24 h dolutegravir plasma concentration-time pharmacokinetic profiling was done in all enrolled children at baseline and 1, 2, 3, 4, 6, and 24 h after observed dolutegravir intake. Target dolutegravir trough concentrations (C_trough_) were based on reference adult pharmacokinetic data and safety was evaluated in all children in the corresponding weight bands who consented to pharmacokinetic studies and received the studied doses.

**Findings:**

Between Sept 22, 2016, and May 31, 2018, we enrolled 62 black-African children aged from 6 years to younger than 18 years (84 pharmacokinetic-profiles). In children weighing 20 kg to less than 25 kg taking 25 mg film-coated tablets, the geometric mean (GM) C_trough_ (coefficient of variation) was 0·32 mg/L (94%), which was 61% lower than the GM C_trough_ of 0·83 mg/L (26%) in fasted adults on dolutegravir 50 mg once-daily; in children weighing 25 kg to less than 30 kg taking 25 mg film-coated tablets, the GM C_trough_ was 0·39 mg/L (48%), which was 54% lower than the GM C_trough_ in fasted adults; and in those 30 kg to less than 40 kg taking 35 mg film-coated tablets the GM C_trough_ was 0·46 mg/L (63%), which was 45% lower than the GM C_trough_ in fasted adults. On 50 mg film-coated tablets or 30 mg dispersible tablets, C_trough_ was close to the adult reference (with similar estimates on the two formulations in children in the 20 to <25 kg weight band), with total exposure (area under the concentration-time curve from 0 h to 24 h) in between reference values in adults dosed once and twice daily, where safety data are reassuring, although maximum concentrations were higher in children weighing 20 kg to less than 25 kg than in the twice-daily adult reference. Over a 24-week follow-up period in 47 children on 30 mg dispersible tablets or 50 mg film-coated tablets, none of the three reported adverse events (cryptococcal meningitis, asymptomatic anaemia, and asymptomatic neutropenia) were considered related to dolutegravir.

**Interpretation:**

Adult dolutegravir 50 mg film-coated tablets given once daily provide appropriate pharmacokinetic profiles in children weighing 20 kg or more, with no safety signal, allowing simplified practical dosing and rapid access to dolutegravir. These results informed the WHO 2019 dolutegravir paediatric dosing guidelines and have led to US Food and Drug Administration approval of adult dosing down to 20 kg.

**Funding:**

Paediatric European Network for Treatment of AIDS Foundation, ViiV Healthcare, UK Medical Research Council.

**Research in context**

**Evidence before this study**

Dolutegravir-based antiretroviral therapy (ART) is being adopted in adults living with HIV globally because it offers better tolerability, fewer adverse drug reactions, fewer drug-drug interactions and higher genetic barrier to resistance than other available ART options; dolutegravir is also accessible for low-income and middle-income countries (LMICs) because of its low cost. Currently, updated WHO guidelines recommend dolutegravir-based ART as preferred first-line regimen for adults living with HIV initiating ART; however, availability and dosing of dolutegravir in children is lagging behind. We searched PubMed for clinical trials, pharmacokinetic, or cohort studies on dolutegravir in paediatric populations (aged >5 years) using the search terms “(pediatric OR paediatric OR children OR adolescents) AND dolutegravir” and relevant conference abstracts up to Oct 15, 2019. The search produced seven articles and two conference abstracts on dolutegravir use in paediatric populations. Two articles and two abstracts were from the ongoing phase 1/2 paediatric dose-finding trial (IMPAACT p 1093), which uses an age-staggered approach to investigate safety and pharmacokinetics for age-appropriate dolutegravir formulations in children aged between 4 weeks and younger than 18 years. The IMPAACT p1093 trial reported intensive pharmacokinetic results, 144-week safety results in children aged 12 years or older (n=23) and 48-week safety results in children aged six and younger than 12 years (n=23). On the basis of results from this trial, stringent regulatory authorities licensed dolutegravir doses for children aged 6 years or older. The US Food and Drug Administration (FDA) licensed dolutegravir for those weighing 30 kg or more and the European Medicines Authority (EMA) approved dolutegravir for those weighing 15 kg or more. Until recently, recommended dosing for children younger than 12 years weighing less than 40 kg was complex, required different paediatric formulations within some weight bands, and there was no alignment for recommended dosing between the FDA and the EMA for children weighing 15 kg to less than 30 kg. Five small observational retrospective European cohort studies evaluated safety and efficacy of dolutegravir in adolescents; one of these also evaluated children and young people aged between 5 years and younger than 12 years (N=33). Observational studies show high viral suppression rates for dolutegravir-based ART in adolescents who are adherent to their ART with only a small number of reported side-effects. Data from real-life cohorts in younger children, aged between 5 years and younger than 12 years, using dolutegravir doses of 20 mg, 25 mg, or 35 mg are scarce.

**Added value of this study**

We nested pharmacokinetic substudies in the ODYSSEY phase 2/3 randomised controlled trial (ClinicalTrials.gov, NCT02259127) aiming to simplify dolutegravir dosing for children using WHO weight bands, which are almost universally used in low-income settings. We evaluated pharmacokinetics and safety by weight band of once-daily dolutegravir dosing using paediatric 10 mg and 25 mg film-coated tablets at the doses approved by the SRAs at the time of the study and simplified dosing with adult 50 mg film-coated tablets in children weighing 20 kg or more, as well as dolutegravir 5 mg dispersible tablets as a dose of 30 mg in children weighing 20 kg to less than 25 kg. The latter is an important alternative option for children unable to swallow adult tablets. 62 children aged between 6 years and younger than 18 years in Uganda and Zimbabwe contributed 84 pharmacokinetic profiles and safety data. The pharmacokinetic results showed that dolutegravir exposures on the SRA-approved doses were lower than adult reference values, whereas once-daily adult dolutegravir 50 mg film-coated tablets in children weighing 20 kg or more and 30 mg dispersible tablets in children weighing 20 kg to less than to 25 kg provided adequate pharmacokinetic profiles with no adverse safety signals.

**Implications of all the available evidence**

The results of pharmacokinetic studies allow implementation of simplified and readily available dolutegravir-based ART for children weighing 20 kg or more using adult tablets. This will enable alignment with adult ART regimens preferred by WHO in LMICs. WHO has included the option of adult dolutegravir film-coated tablets for children weighing more than 20 kg in their updated 2019 guidelines. Based on the ODYSSEY data the FDA has recently approved the 50 mg film-coated adult tablet and 30 mg dispersible tablets for children weighing20 kg or more.

## Introduction

Approximately 1·7 million children younger than 15 years are living with HIV worldwide, and in 2018, about 160 000 children newly acquired HIV—of those, approximately 90% were from sub-Saharan Africa.^1^, ^2^ These children and adolescents require lifelong antiretroviral therapy (ART) to inhibit viral replication, to prevent HIV-related morbidity and mortality, and to stop transmission of the virus to others. However, in 2018, only an estimated 56% of children with HIV had access to ART,^1^ and treatment outcomes in children and adolescents were consistently worse than in adults.^3^ Endorsed by recently updated WHO guidelines,^4^ several countries are rolling out dolutegravir, an integrase strand transfer inhibitor, as the preferred third ART drug combined with two nucleoside reverse transcriptase inhibitors for first-line treatment across all ages.^5^ In adults, dolutegravir is highly effective in rapidly reducing HIV-1 RNA viral load and has a high barrier to development of resistance. Additionally, dolutegravir has a favourable safety profile with few drug-drug interactions.^6^ ART regimens, including dolutegravir, are low cost and already available in low-income and middle-income countries (LMICs) as well as high-income countries.^5^ A key WHO principle for treatment optimisation is harmonisation across populations making dolutegravir the strategically preferred choice.^4^

For children in LMICs, optimised treatment options lag behind those for adults.^7^ On the basis of results from IMPAACT P1093 (NCT01302847), a paediatric phase 1/2 dose-finding trial,^8^, ^9^ Stringent Regulatory Authorities (SRAs) have approved once-daily dolutegravir 50 mg in film-coated tablet form, which is the dose and delivery method for adults, for use in adolescents aged 12 years and older weighing 40 kg or more. Until recently, once-daily dolutegravir 35 mg in film-coated tablet form (given as one 25 mg tablet plus one 10 mg tablet) was approved by the US Food and Drug Administration (FDA) for children weighing 20 kg to less than 30 kg. The European Medicines Agency and SRAs in Canada and Australia also approved once-daily dolutegravir 25 mg in film-coated tablet form for children weighing 20 kg to less than 30 kg and once-daily dolutegravir 20 mg in film-coated tablet form for children weighing 15 kg to less than 20 kg.^10^, ^11^ The paediatric dolutegravir formulations (10 mg and 25 mg film-coated tablets) are not available in most LMICs and the dosing recommendations are therefore impractical. The paediatric antiretroviral market is small and fragmented, and stock-outs in low-level health facilities are frequent.^12^ Additionally, children in the lower weight bands would require yet another (dispersible) formulation.^13^, ^14^ Last, complex regimens with two different tablet doses of the same drug have the potential to cause dosing errors by both health-care workers and caregivers.^15^

Until very recently there was no approved dolutegravir dosing for infants and no consensus on dosing in children weighing 15–30 kg and options for first-line and second-line ART regimens for children failing protease inhibitor-based ART were scarce. Simplified and pragmatic dosing with an alternative robust and tolerable antiretroviral agent, such as dolutegravir, is therefore urgently required for both first-line and second-line treatment for children.

Within the phase 2/3 paediatric ODYSSEY trial (ClinicalTrials.gov, CT02259127), we did nested pharmacokinetic substudies to evaluate the pharmacokinetics and safety for a practical paediatric dolutegravir dosing approach, according to WHO weight bands, and using a minimal number of formulations and doses. We present the results of three intensive pharmacokinetic substudies in children with HIV weighing 20 kg to less than 40 kg.

## Methods

### Study design and participants

ODYSSEY is an open-label, multicentre, randomised, non-inferiority, phase 2/3 trial evaluating efficacy and safety of dolutegravir plus two nucleoside reverse transcriptase inhibitors versus standard of care over 96 weeks follow-up in 700 children with HIV (younger than 18 years), starting first-line or second-line ART in Africa, Europe, and Thailand. We did pharmacokinetic substudies in four ODYSSEY sites in Uganda and Zimbabwe (University of Zimbabwe Clinical Research Centre, Harare, Zimbabwe; Joint Clinical Research Centre, Mbarara, Uganda; Joint Clinical Research Centre, Kampala, Uganda; Baylor College of Medicine Children's Foundation, Kampala, Uganda) and approved by local ethics committees.

The pharmacokinetic substudy for children weighing 25 kg to less than 40 kg was a within-subject, two-period, fixed-order, pharmacokinetic study with paediatric dolutegravir doses approved by the SRAs at the time of the study used in the first period and a higher (adult) dose of 50 mg as film-coated tablet in the second period. A child first completed 24-h intensive pharmacokinetic sampling after at least 1 week of receiving 25 mg film-coated tablets if they were in the 25 kg to less than 30 kg weight band or 35 mg (one 10 mg and one 25 mg film-coated tablet) if they were in the 30 to less than 40 kg weight band. The second 24-h pharmacokinetic profile was taken at least 1 week after the switch to a daily 50 mg film-coated tablet.

In a parallel pharmacokinetic substudy, children weighing 20 kg to less than 25 kg had 24-h intensive pharmacokinetic profiling on the once-daily dose of dolutegravir 25 mg film-coated tablet (part one). After review of the results, we did a subsequent substudy (part two) in children weighing 20 to less than 25 kg, investigating higher dolutegravir doses to achieve better exposures. On the basis of results of part one and to simplify dosing and future access to formulations, we studied the 50 mg adult film-coated tablet. Because we were concurrently investigating dispersible dolutegravir tablets in children weighing less than 20 kg (not reported here), we also studied a 30 mg dose given as six 5 mg dispersible tablets, which is bioequivalent to a 50 mg film-coated tablet,^16^ as an option for children unable to swallow adult tablets. Dolutegravir 50 mg film-coated tablet or 30 mg dispersible tablet formulations were allocated by site. This substudy primarily enrolled children who had not participated in part one because children initially recruited in this weight band had gained weight and were no longer in the weight band.

We asked carers and children for additional informed consent (and assent respectively when appropriate) to participate in the pharmacokinetic substudies until at least eight children per weight band, dose, and formulation, had evaluable pharmacokinetic results. According to Wang and colleagues^17^ a power of 80% is reached with eight individuals and a coefficient of variation of 31–35%. We excluded children with symptoms that could affect pharmacokinetic results (ie, severe diarrhoea, vomiting, renal, or liver disease), severe malnutrition, or taking concomitant medication known to have drug-drug interactions with dolutegravir.

### Procedures

Children enrolled in the pharmacokinetic substudies attended study visits following the main ODYSSEY trial protocol (visits every 12 weeks after week 4 from enrolment). Participants whose dose was increased to 30 mg dispersible tablets or the 50 mg film-coated tablet had additional safety visits at weeks 2, 4, and 12 after dose adaptation. At each visit, we did a clinical assessment, including haematology and biochemistry safety parameters. We ascertained serious adverse events (defined according to International Conference on Harmonisation Guideline for Good Clinical Practice), grade 3 and 4 clinical and laboratory events, and events resulting in modification of ART (any grade).

To achieve steady-state, children attended their pharmacokinetic study visit day at least 7 days after starting on the investigated dolutegravir dose. We ascertained medication adherence during the last 3 days before the day of pharmacokinetic assessment to ensure that no doses had been missed. To construct a dolutegravir plasma concentration-time profile, blood samples of 2 mL were taken at baseline and 1, 2, 3, 4, 6, and 24 h after observed dolutegravir intake with 100 mL of water. To conform with adult comparator studies, participants fasted at least 3 h before dolutegravir intake, although an overnight fast was preferred. Dispersible tablets were dispersed in a small amount of water and immediately taken, followed by an additional intake of water up to a volume of 100 mL. We allowed no medications, other than nucleoside reverse transcriptase inhibitors, within 2 h after dolutegravir intake. Children fasted 2 h after dolutegravir intake before having a light breakfast (containing approximately 250 calories). During pharmacokinetic assessment days all food was provided at set times.

We refrigerated blood samples within 10 min and centrifuged them within 24 h of collection. Plasma was separated and stored at −80°C until shipping to the laboratory of the Department of Pharmacy, Radboudumc, Nijmegen, Netherlands for quantification with a validated liquid chromatography tandem mass spectrometry bioanalytical quantification method (lower limit of quantification of 0·01 mg/L).^18^

### Statistical analysis

We included all participants consenting to inclusion in the pharmacokinetic substudies in the safety analyses. For the pharmacokinetic analyses, we considered haemolysed samples non-evaluable and excluded pharmacokinetic profiles if treatment non-adherence was likely (predefined when a predose concentration [C_0_] was >15 times below the concentration 24 h after observed drug intake (trough concentration [C_trough_]), if protocol violations had occurred, or if concomitant medications known to affect dolutegravir drug concentrations had been used.

We used Phoenix WinNonlin 64 (version 8.1; Certara, Princeton, NJ, USA) for non-compartmental analysis and calculation of summary statistics for dolutegravir pharmacokinetic parameters; C_trough_, C_0_ (predose sample), area under the concentration-time curve from 0 to 24 h (AUC_0–24h_), maximum plasma concentration (C_max_), apparent elimination half-life (T_1/2_), oral clearance (CL/F), and volume of distribution (Vd/F). C_0_, C_max,_ and obtained the time to the maximum plasma concentration (T_max_) directly from the observed concentration-time data. We estimated C_trough_ using T_1/2_ if the sample was not taken at 24 h (four curves).

The aim was to achieve pharmacokinetic parameters comparable to published values for approved adult dolutegravir dosing.^19^, ^20^, ^21^ In particular, we aimed to record similar geometric mean (GM) C_trough_ to adult GM C_trough_ on dolutegravir 50 mg film-coated tablets given once daily under fasted conditions. We considered dolutegravir C_trough_ as the main pharmacokinetic target, as it correlates with viral load decline and is linked to antiviral response.^19^, ^22^ We also compared pharmacokinetic results to published adult pharmacokinetic reference data on the dolutegravir 50 mg film-coated tablet given twice daily, because this is the highest approved dose that has a reassuring safety profile. We considered the proportion of individuals with C_trough_ lower than 0·32 mg/L, which equals the in vivo dolutegravir 90% effective concentration (EC_90_; the effective concentration at which 90% of maximal viral inhibition is achieved in a 10-day monotherapy study).^19^ Data on HIV-1 RNA viral loads were not available for this analysis because virological failure is a component of the primary endpoint in the main ODYSSEY trial, which is still ongoing. For estimation of GM ratios (GMRs) and 90% CIs for paired and non-paired pharmacokinetic data on different dolutegravir doses and formulations we used a linear mixed-model (with dose as fixed-effect and random effect for participant) or an independent *t* test as appropriate on log-transformed pharmacokinetic parameters with IBM Statistical Package for the Social Sciences (version 25). We assessed dose-proportionality for log-transformed and dose normalised pharmacokinetic parameters (AUC_0–24h_ and C_max_) for adult dolutegravir 50 mg film-coated tablet doses versus doses approved by the SRAs at the time of the study. In the weight band 20 kg to less than 25 kg, we assessed equivalence in exposures for the adult 50 mg film-coated tablet versus paediatric 30 mg dispersible tablets. In both situations we used the standard 0·80–1·25% equivalence limits that are acknowledged by the SRAs to decide whether dose-linearity or equivalence was shown following criteria as defined by Williams and colleagues*.*^23^

All children consenting to inclusion in a pharmacokinetic substudy contributed safety data from the start of each pharmacokinetic dose for 24 weeks or to last follow-up visit, whichever was earlier; no participant commenced a second pharmacokinetic dose before completing 24 weeks on the first pharmacokinetic dose with the exception of children weighing 25 kg to less than 40 kg crossing over from the doses approved by the SRAs at the time of the study the 50 mg film-coated tablet, with which follow-up could be shorter. As part of the ODYSSEY trial, an independent endpoint review committee who were masked to trial groups (third drug of ART regimen) reviewed all reported adverse events. We did descriptive analysis reporting follow-up and adverse events by dose and weight band in STATA (version 15.0). An independent data monitoring committee reviewed conduct, pharmacokinetic, and safety data from these substudies. The ODYSSEY trial is registered with ClinicalTrials.gov, NCT02259127.

### Role of the funding source

The ODYSSEY trial is sponsored by the Paediatric European Network for Treatment of AIDS Foundation. The Medical Research Council Clinical Trials Unit at University College London, London, UK receives core support from the UK Medical Research Council (grant number MC_UU_12023/26). The Paediatric European Network for Treatment of AIDS Foundation provides support to sites in Europe. This study received funding from ViiV Healthcare, who reviewed and provided comments on the manuscript, although the authors of the Article were responsible for the analysis and interpretation of the data. The corresponding author had full access to all the data in the study and had final responsibility for the decision to submit for publication.

## Results

Between Sept 22, 2016, and May 31, 2018, we enrolled 62 black-African children, aged from 6 years to younger than 18 years, and included all in the safety population. Most children (33 [73%] of 45) had no dolutegravir intake before starting the doses of 25 mg film-coated tablet and 35 mg film-coated tablets; by contrast, all children starting 30 mg dispersible tablets or 50 mg film-coated tablet had taken dolutegravir previously for a median of 33 weeks (range 2–61; table 1).

**Table 1:** Patient demographics and characteristics by weight band at time of dolutegravir dose initiation

		**20 kg to <25 kg (n=34)**			**25 kg to <30 kg (n=17)**		**30 kg to <40 kg (n=11)**	
		25 mg FCT	30 mg DT	50 mg FCT	25 mg FCT	50 mg FCT	35 mg FCT	50 mg FCT
Number of children consenting to participate		17	10*	9*	17	17	11	11
Ethnicity, black-African		17 (100%)	10 (100%)	9 (100%)	17 (100%)	17 (100%)	11 (100%)	11 (100%)
Sex								
	Male	9 (53%)	6 (60%)	6 (67%)	13 (76%)	13 (76%)	4 (36%)	4 (36%)
	Female	8 (47%)	4 (40%)	3 (33%)	4 (24%)	4 (24%)	7 (64%)	7 (64%)
Age, years (median [IQR, range])		9·5 (7·6–10·5; 6·9–11·5)	7·6 (7·3–9·5; 5·8–11·3)	9·0 (8·1–9·7; 5·9–11·7)	10·4 (9·1–11·0; 7·2–15·4)	10·7 (9·7–11·5; 7·5–15·9)	10·9 (9·9–12·3; 9·8–17·7)	11·3 (10·3–12·6; 9·8–17·8)
Weight, kg (median [IQR; range])		22·5 (21·2–23·3; 20·3–25·4)	21·3 (20·5–22·4; 20·0–22·9)	22·0 (21·0–23·0; 20·4–24·5)	26·4 (25·6–26·9; 25·0–28·4)	27·4 (25·8–28·5; 25·0–30·7)†	30·2 (30·0–31·4; 28·5–37·6)‡	31·0 (30·2–36·0; 29·9–38·7)§
Previous ART experience								
	Receiving first-line ART	3 (18%)	6 (60%)	0 (0%)	9 (53%)	9 (53%)	7 (64%)	7 (64%)
	Receiving second-line ART	14 (82%)	4 (40%)	9 (100%)	8 (47%)	8 (47%)	4 (36%)	4 (37%)
Previous dolutegravir exposure, weeks		0·0 (0·0–0·0; 0·0–0·0)	34·6 (21·7–40·7; 13·9–60·7)	47·1 (34·4–48·0; 24·6–60·0)	0·0 (0·0–4·0; 0·0–35·9)	28·0 (18·0–36·1; 8·0–51·0)	1·0 (0·0-24·0; 0·0–36·0)	23·0 (13·1–37·7; 2·1-44·0)

Data are n (%) or median (IQR), unless otherwise stated. FCT=film-coated tablet. DT=dispersible tablets. ART=antiretroviral therapy.

* Two participants in weight band between 20 kg and lower than 25 kg contributed safety data on dolutegravir 25 mg FCT (part one) and subsequently on 50 mg FCT (n=1) or 30 mg DT (n=1) in part two.

† One participant increased in weight at the start of 50 mg FCT to 30·7 kg (previously within 25 to <30 kg, receiving 25 mg FCT).

‡ Two participants started 35 mg FCT weighing 28·5 kg and 29·9 kg.

§ One participant started 50 mg FCT weighing 29·9 kg (previously within 30 to <40 kg weight band, receiving 35 mg FCT). By the time of their pharmacokinetic assessment day their weight had increase to more than 30 kg.

**Figure 1:**
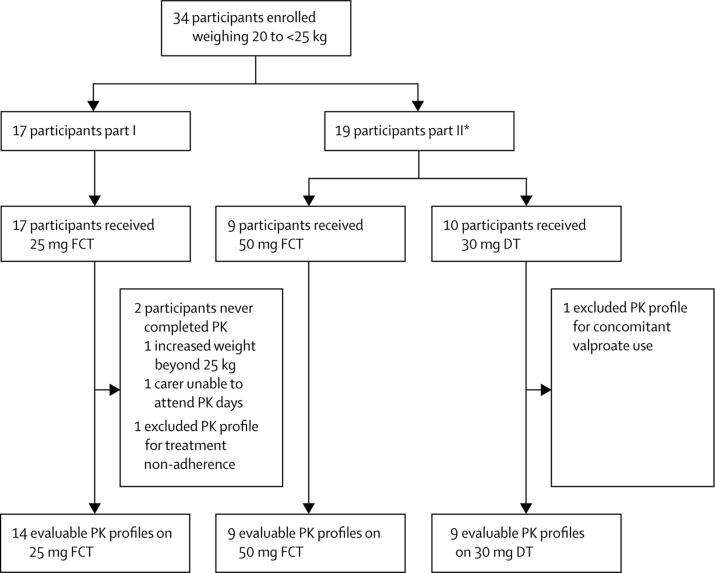
Substudy profile for cohorts weighing 20 kg to less than 25 kg Non-adherence was predefined as a trough concentration-predose concentration ratio of more than 15. FCT=film-coated tablet. DT=dispersible tablets. PK=pharmacokinetic. *Two participants in the 20 kg to less than 25 kg weight band contributed safety data on 25 mg FCT in part one and subsequently on 50 mg FCT (n=1) or 30 mg dispersible tablets (n=1) in part two.

**Figure 2:**
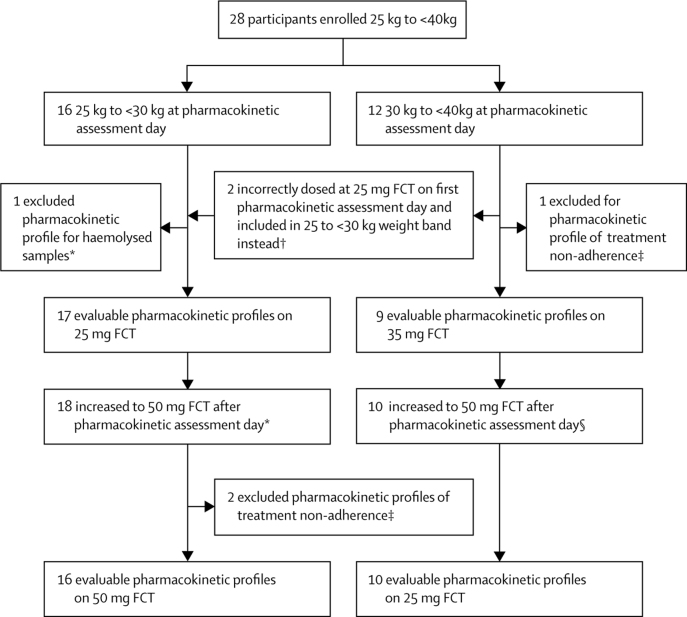
Substudy profile for cohort weighing 25 kg to less than 40 kg FCT=film coated tablet. *The child with haemolysed samples on the 25mg FCT had a valid pharmacokinetic curve on the increased dose of 50 mg FCT. †One participant (30·6 kg at pharmacokinetic assessment day) was dosed at 25 mg, although slightly out of weight band and included in the weight band 25 kg to less than 30 kg for pharmacokinetics and safety analyses. One participant (30·7 kg at the pharmacokinetic assessment day) was on 35 mg FCT but was given 25 mg FCT at the pharmacokinetic assessment day only and included in the weight band 25 kg to less than 30 kg for pharmacokinetic analyses and in weight band 30 kg to less than 40 kg for safety analyses. ‡Pharmacokinetic non-adherence predefined as a trough concentration-predose concentration ratio of more than 15. §For one child the pharmacokinetic profile while on 35 mg was excluded from pharmacokinetic analysis. The pharmacokinetic profile while on 50 mg from the same child showed no signs of non-adherence and was included in the pharmacokinetic analysis.

**Figure 3:**
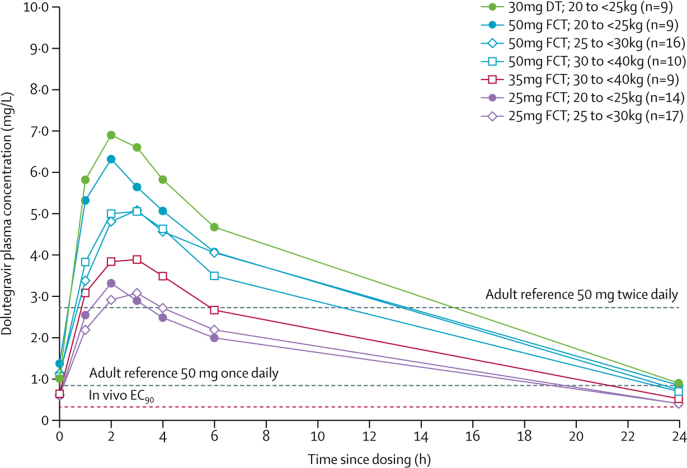
Dolutegravir mean plasma concentration versus time profiles by weight band and dose 30 mg dispersible tablet was given as six 5 mg DTs. 50 mg was a single film-coated tablet. 35 mg FCT was given as one 25 mg FCT plus one 10 mg FCT. 25 mg was given as a single film-coated tablet. Adult reference lines (grey) indicate published geometric mean trough concentrations for 50 mg once daily or twice daily. Orange horizontal line expresses the in vivo EC90 (0·32 mg/L). FCT=film-coated tablets. DT=disperisble tablets. EC90=effective concentration at which 90% of maximal viral inhibition is achieved.

Across the described pharmacokinetic substudies, we included 58 (94%) of 62 children in the pharmacokinetic population and they contributed to 84 pharmacokinetic profiles (figure 1, figure 2). Figure 3 depicts mean plasma concentration versus time profiles for all doses by weight band. In children weighing 20 kg to less than 25 kg taking 25 mg film-coated tablets, the GM C_trough_ (coefficient of variation) was 0·32 mg/L (94%), which was 61% lower than the GM C_trough_ of 0·83 mg/L (26%) in fasted adults on dolutegravir 50 mg once-daily; in those weighing 25 kg to less than 30 kg taking 25 mg film-coated tablets, the GM C_trough_ was 0·39 mg/L (48%), which was 54% lower than in fasted adults; and in those weighing 30 kg to less than 40 kg taking 35 mg film-coated tablets the GM C_trough_ was 0·46 mg/L (63%), which was 45% lower than in fasted adults (table 2).

**Table 2:** Summary of dolutegravir pharmacokinetic parameters in children by weight band and published adult reference pharmacokinetic parameters

	**20 kg to <25 kg**			**25 kg to 30 kg**		**30 kg to <40 kg**		**Adults ≥40 kg**^19^	**Adults ≥40 kg**^20^, ^21^
	25 mg FCT	30 mg DT	50 mg FCT	25 mg FCT	50 mg FCT	35 mg FCT	50 mg FCT	50 mg FCT	50 mg BID FCT
Number of participants with pharmacokinetic profiles	14	9	9	17	16	9	10	10*	24†
Age on pharmacokinetic assessment day, years	9·5 (7·6–10·6)	7·8 (7·5–9·5)	9·1 (8·1–9·7)	10·8 (9·7–11·9)	10·5 (9·3–11·4)	11·3 (10·5–12·5)	11·3 (10·4–12·5)	..	..
Weight on pharmacokinetic assessment day, kg (IQR; range)	23·4 (22·9–23·9; 20·2–24·3)	21·9 (21·0–22·5; 20·2–22·7)	21·6 (20·6–23·0; 20·2–24·5)	27·3 (26·5–28·7; 25·0–30·7)‡§	27·9 (26·7–29·5; 25.4–30·6)‡§	31·0 (30·1–35·0; 29·9–38·2)¶	31·6 (30·3–37·1; 30·0–38·5)	..	..
Dose, mg/kg	1·1 (1·0–1·1)	1·4 (1·3–1·4)	2·3 (2·2–2·4)	0·9 (0·9–0·9)	1·8 (1·7–1·9)	1·1 (1·0–1·2)	1·6 (1·3–1·7)	..	..
Previous dolutegravir exposure on pharmacokinetic day, weeks	7·5 (2·0–19·4)	34·9 (23·7–40·6)	48·3 (36·1–51·7)	27·9 (17·9–36·0)	30·4 (19·9–40·2)	23·1 (16·9–37·6)	24·6 (16·9–39·6)	..	..
Previous exposure to investigated pharmacokinetic dose on pharmacokinetic assessment day, weeks (IQR; range)	7·5 (2·0–19·4; 1·6–48·0)	1·9 (1·7–2·0; 1·3–2·6)	1·7 (1·3–2·1; 1·1–3·7)	18·9 (14·6–29·9; 0·0–45·9)‖	1·9 (1·9–1·9; 1·0–5·4)	14·9 (7·9–22·9; 2·0–37·6)	1·9 (1·9–1·9; 1·0–1·9)	..	..
C_0_, mg/L	0·49 (80)	0·85 (76)	1·26 (49)	0·53 (59)	1·05 (45)	0·51 (97)	1·00 (37)	..	3·20 (69)
C_trough_, mg/L	0·32 (94)	0·76 (73)	0·75 (44)	0·39 (48)	0·77 (43)**	0·46 (63)	0·63 (49)	0·83 (26)	2·72 (70)
Proportion of patients below EC_90_	4 (29%)	1 (11%)	0 (0%)	5 (29%)	0 (0%)	3 (33%)	0 (0%)		
AUC_0–24h_, h × mg/L	30·0 (41)	71·4 (26)	63·7 (26)	33·1 (23)	58·6 (28)**	40·3 (35)	53·5 (32)	43·4 (20)	93·4 (50)
C_max_, mg/L	3·20 (40)	7·16 (26)	6·34 (27)	3·16 (24)	5·41 (25)	3·98 (28)	5·22 (25)	3·34 (16)	5·41 (40)
T_max,_h	2·0 (1·0–3·0)	3·0 (2·0–4·0)	2·0 (1·0–3·0)	2·0 (2·0–4·0)	3·0 (2·0–6·0)	3·0 (1·0–4·0)	2·5 (2·0–4·0)	2·0 (1·0–4·0)	2·0 (0–7·9)
T_1/2_, h	7·65 (51)	7·14 (30)	7·49 (19)	7·26 (23)††	8·14 (16)‡‡	6·85 (17)§§	7·45 (15)¶¶	12·0 (22)	..
CL/F, L/h	0·83 (41)	0·42 (26)	0·79 (26)	0·76 (23)**	0·85 (28)**	0·87 (35)	0·93 (32)	..	..
Vd/F, L	9·19 (62)	4·33 (25)	8·48 (28)	7·94 (27)††	9·66 (29)‡‡	8·61 (31)§§	10·1 (29)¶¶	..	..

Data are geometric mean (coefficient of variation %), median (IQR), or n (%), unless indicated otherwise. FCT=film-coated tablet. DT=dispersible tablets. BID=twice daily. C_0_=predose concentration. C_trough_=trough concentration. EC90= the effective concentration at which 90% of maximal viral inhibition is achieved in a 10-day monotherapy study. AUC_0–24h_=area under the concentration-time curve from 0 to 24 h. C_max_=maximum plasma concentration. T_max_=time to maximum plasma concentration. T_1/2_=apparent elimination half-life. CL/F=oral clearance. Vd/F=volume of distribution.

* Fasted adults who were HIV-positive.

† Adults who were HIV-positive and had previous treatment, fed state not specified.

‡ One participant received 25 mg FCT while weighing 30·6 kg and was included in the weight band between 25 kg and lower than 30 kg; weight was the same (30·6 kg) on the second pharmacokinetic assessment day on 50 mg FCT; therefore, the pharmacokinetic profile was also included in the results for the weight band between 25 kg and less than 30 kg for their second pharmacokinetic assessment day.

§ One participant started on 35mg FCT but was accidentally administered 25mg FCT for their first pharmacokinetic day, weight was 30·7kg and the pharmacokinetic profile was included in the 25 to less than 30kg weight band for the first pharmacokinetic day. There was no pharmacokinetic profile available on the 50mg dose. This participant is considered within safety follow-up for the 30 to less than 40kg group.

¶ One participant on 35 mg FCT while weighing 29·9 kg was included in the weight band between 30 kg and less than 40 kg; weight on the second pharmacokinetic assessment day on 50 mg FCT was 30·1 kg.

‖ One participant started on 35 mg FCT, but was accidentally administered 25 mg FCT only on the pharmacokinetic assessment day (see § above). Exposure to 35 mg FCT before pharmacokinetic assessment day was 13·0 weeks; another participant started on 25 mg while weighing between 20 kg and less than 25 kg, their weight increased to more than 25 kg the day before the pharmacokinetic assessment day, exposure to 25 mg while weighing between 20 kg and less than 25 kg was 3 weeks; the range for previous exposure to the investigated pharmacokinetic dose on the pharmacokinetic assessment day, excluding these two participants, is 7·9–45·9 weeks.

** n=15.

†† n=16.

‡‡ n=12.

§§ n=8.

¶¶ n=9.

**Figure 4:**
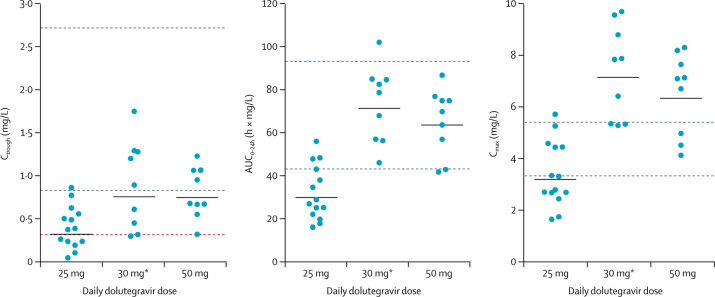
Individual dolutegravir C_trough_, AUC_0–24h_, and C_max_ in children weighing 20 kg to less than 25 kg Horizontal black lines indicate geometric means per dose. Orange line indicates dolutegravir in-vivo EC_90_. Grey dashed lines indicate published geometric mean adult reference values for 50 mg once-daily (lower line) and twice-daily doses (upper line). C_trough_=trough concentration. AUC_0–24h_=area under the concentration-time curve from 0 to 24 h. C_max_=maximum concentration. EC_90_=the effective concentration at which 90% of maximal viral inhibition is achieved in a 10-day monotherapy study. *Dispersible tablets.

**Figure 5:**
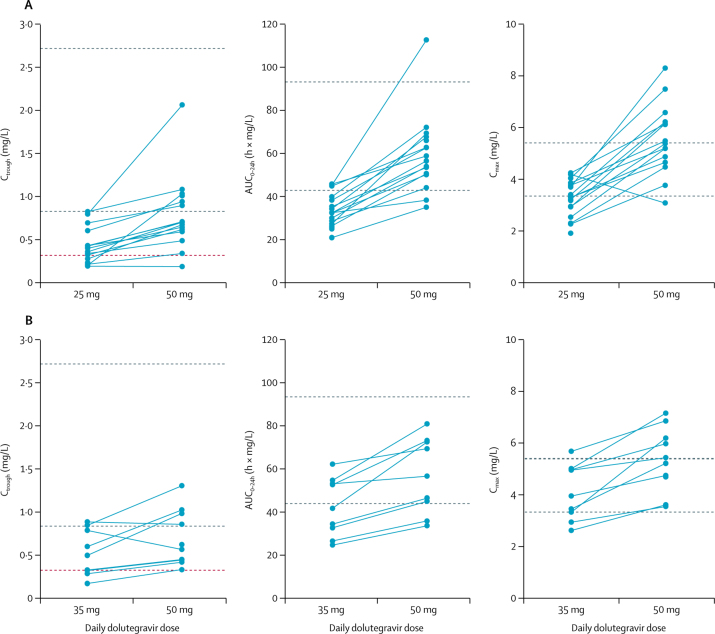
Individual dolutegravir C_trough_, AUC_0–24h_, and C_max_ on the initial dolutegravir dose and after switch to adult formulation Children weighing 25 kg to less than 30 kg (A). Children weighing 30 kg to less than 40 kg (B). Grey dashed lines indicate published geometric mean adult reference values for 50 mg once-daily (lower line) and twice-daily doses (upper line). Red dashed line indicates the effective concentration at which 90% of maximal viral inhibition is achieved in a 10-day monotherapy study (EC_90_). C_trough_=trough concentration. AUC_0–=24h_=area under the concentration-time curve from 0 to 24 h. C_max_=maximum concentration.

12 (30%) of 40 children who took the doses approved by the SRA at the time of the study had individual C_troughs_ below 0·32 mg/L, the dolutegravir in vivo EC_90_. By contrast, the children weighing 20 kg to less than 25 kg who took adult 50 mg film-coated tablets had GM C_trough_ of 0·75 mg/L (44%), those weighing 25 kg to less than 30 kg had GM C_trough_ of 0·77 mg/L (43%), and those weighing 30 kg to less than 40 kg had GM C_trough_ of 0·63 mg/L (49%), which were similar to adult reference values, and none had a C_trough_ below the dolutegravir EC_90_. The GM C_trough_ in children taking 30 mg dispersible tablets was 0·76 mg/L (73%), which was similar to C_trough_ on 50 mg once daily in adults, and only one child taking 30 mg dispersible tablets had C_trough_ below EC_90_ (table 2, figure 4, figure 5).

Total dolutegravir exposure (expressed as AUC_0–24h_) in children weighing 20 kg or more taking 50 mg film-coated tablets (or 30 mg dispersible tablets in children weighing 20 kg to <25 kg) was above the reference AUC_0–24h_ in adults taking 50 mg once-daily, but did not exceed the reference for twice-daily dosing. However, GM C_max_ in children weighing 20 kg to less than 25 kg were 17% higher on the 50 mg film-coated tablet and 32% higher on 30 mg dispersible tablets than in adults on 50 mg twice-daily (figure 4).

We observed a dose-proportional increase in exposure, based on AUC_0–24h_ and C_max_, for the 50 mg film-coated tablet compared with the 25 mg and 35 mg film-coated tablets (appendix p 1, table 1). Additionally, daily dosing of 50 mg film-coated tablet versus 30 mg dispersible tablets in children weighing 20 kg to less than 25 kg resulted in similar AUC_0–24h_ and Cmax: GMR 0·89 (90% CI 0·70–1·15) and 0·89 (90% CI 0·68–1·15) respectively (appendix p 1, table 2).

After a follow-up of 24 weeks in all 17 children weighing 20 kg to less than 25 kg receiving 25 mg film-coated tablets dose, two asymptomatic neutropenias (grade 3 laboratory adverse events) and one asymptomatic thrombocytopenia with raised transaminases (grade 3 laboratory adverse event) occurred (table 3). After a median follow-up of 19 weeks (IQR 15–24) in 17 children weighing 25 to less than 30 kg receiving 25 mg film-coated tablets, and after 13 weeks (6–23) in 11 children weighing 30 kg to less than 40 kg receiving 35 mg film-coated tablets, no reportable adverse events occurred.

**Table 3:** Summary of adverse events by weight band and dolutegravir dose and formulation

			**20 to <25 kg (n=34)**			**25 to <30 kg (n=17)**		**30 to <40 kg (n=11)**	
			17 patients (25 mg) FCT	10 patients (30 mg)* DT	9 patients (50 mg)* FCT	17 patients (25 mg) FCT	17 patients (50 mg) FCT	11 patients (35 mg) FCT	11 patients (50 mg) FCT
Follow-up time, weeks			24·0 (24·0–24·0)†	24·0 (24·0–24·0)	24·0 (24·0–24·0)	19·0 (15·1–24·0)‡	24·0 (24·0–24·0)§	13·1 (6·3–23·0)¶	24·0 (24·0–24·0)§
Total follow-up time, weeks			408	223	189	304	408	145	264
Total adverse events‖			3 (18%)	0	0	0	1 (6%)	0	2 (18%)
	Grade 3 or 4 clinical adverse events		0	0	0	0	0	0	1 (9%)
		Cryptococcal meningitis	0	0	0	0	0	0	1 (9%)**
	Grade 3 or 4 asymptomatic laboratory events		3 (18%)	0	0	0	1 (6%)	0	1 (9%)
		Anaemia	0	0	0	0	0	0	1 (9%)
		Neutropenia	2 (12%)	0	0	0	1 (6%)	0	0
		Thrombocytopenia and raised transaminases	1 (6%)	0	0	0	0	0	0

Data are n (%) and median (IQR), unless otherwise stated. FCT=film-coated tablet. DT=dispersible tablet.

* Two participants in weight band between 20 kg and less than 25 kg contributed safety data on dolutegravir 25 mg FCT (part one) and subsequently on 50 mg FCT (n=1; part two) or 30 mg DT (n=1; part two).

† One participant received twice-daily dolutegravir (for use of rifampicin) for the total duration (24 weeks) of safety follow-up for 25 mg FCT dose. No adverse events occurred within this period.

‡ Two participants received twice-daily dolutegravir (for use of rifampicin) for the total duration (24 weeks) of safety follow-up for 25 mg FCT dose. No adverse events occurred within this period for either participant. During follow-up the protocol required twice-daily dolutegravir (on current dose) until 2 weeks after stopping rifampicin. Rifampicin as an interacting co-medication was not allowed during the pharmacokinetic evaluations.

§ One participant in the 30 kg to less than 40 kg weight band was incorrectly dosed 35 mg FCT for 1 day and one participant in the 25 kg to less than 30 kg weight band was incorrectly dosed 25 mg FCT for 84 days, while within the safety follow-up period for 50 mg FCT. No adverse events occurred within this period for either participant.

¶ One participant weighing between 30 kg and less than 40 kg was incorrectly administered 25 mg for 1 day (pharmacokinetic assessment day) while within the safety follow-up period for the 35 mg FCT.

‖ Grade 3 or 4 or serious-adverse event. No events led to a change in antiretroviral therapy and none were considered by an independent Endpoint Review Committee to be related to dolutegravir.

** Serious adverse event.

After a median follow-up of 24 weeks (IQR 24–24) in ten children weighing 20 kg to less than 25 receiving 30 mg dispersible tablets and a median follow-up of 24 weeks (24–24) in nine children weighing 20 kg to less than 25 kg who took 50 mg film-coated tablets, there were no reportable adverse events (table 3). After a follow-up of 24 weeks in all 28 children weighing 25 kg or more receiving 50 mg film-coated tablets, there were three reportable adverse events. These included one serious adverse event, which was cryptococcal meningitis (WHO stage 4 and Division of AIDS grade 4), one asymptomatic anaemia (grade 3 laboratory event) and one asymptomatic neutropenia (grade 3 laboratory event).

Over a total follow-up of 20·5 patient-years in 47 children on either the 50 mg film-coated tablet or 30 mg dispersible tablet, there were 3 adverse events reported (rate 14·6/100 person years [95% CI 3·1–43]; table 3). The reporting clinicians and endpoint review committee considered none of the adverse events to be related to dolutegravir exposure and no events resulted in modification of ART.

## Discussion

These ODYSSEY pharmacokinetic substudies showed that taking the once-daily dolutegravir 50 mg film-coated tablet resulted in appropriate drug exposures in children weighing 20 kg or more. In addition, 30 mg dispersible tablets, evaluated in children weighing 20 kg to less than 25 kg, offer a practical alternative for children weighing 20 kg or more unable to swallow tablets. Safety signals were not seen for any of the doses studied.

An accepted approach to providing evidence supporting the use of drugs in children is to determine a paediatric dose that shows drug exposure sufficiently similar to the exposure that is effective and safe in adults. Dolutegravir clinical studies showed that minimum plasma concentrations correlated with reduction in HIV-1 viral load after 10-day monotherapy^19^ and with virological outcomes after 48 weeks of treatment in a treatment-experienced population.^6^, ^22^ Therefore, we chose dolutegravir C_trough_ as the main target pharmacokinetic parameter. Our results show that GM trough concentrations in children 20 kg to less than 40 kg on dolutegravir film-coated tablets licensed at the time of the study were lower than published reference values in adults on dolutegravir 50 mg once daily;^19^ although there are no data showing that paediatric dosing with 20 mg, 25 mg, or 35 mg are associated with virological failure or resistance. On the increased doses (50 mg film-coated tablet for the weight band 20 kg to less than 40 kg and 30 mg dispersible tablets for the weight band 20 kg to less than 25 kg) C_trough_ concentrations were reassuringly similar to the adult references.

Initial paediatric dolutegravir doses were previously licensed on the basis of pharmacokinetic and safety data from the ongoing phase 1/2 dose-finding IMPAACT P1093 trial, evaluating safety, tolerability, and pharmacokinetics for age-appropriate dolutegravir formulations. Intensive pharmacokinetic sampling within the IMPAACT P1093 trial showed GM C_trough_ concentrations (0·93 mg/L) in children aged 6 years to younger than 12 years to be similar to those in adults on film-coated tablets: weight-based dosing of approximately 1 mg/kg; 50 mg in children weighing more than 40 kg (n=5), 35 mg in children weighing 30 kg to less than 40 kg (n=2), and 25 mg in children weighing 20 kg to less than 30 kg (n=4). These data led to approval of the 35 mg film-coated tablet dose for children weighing 30 kg to less than 40 kg by the SRAs. The proposed 25 mg film-coated tablet dose in children weighing 20 kg to less than 30 kg was also approved by European Medicines Agency and the SRAs in Australia and Canada but not by the FDA. A subanalysis by the FDA revealed lower GM C_trough_ (0·52 mg/L) in children weighing 20 kg to less than 30 kg (n=4) than in the adult reference.^22^, ^24^

The minimal dolutegravir trough concentration to attain optimal viral inhibition in vivo is not yet fully elucidated. Although the in-vitro protein adjusted minimal inhibitory concentration (IC_90_) for dolutegravir was established at 0·064 mg/L,^25^ the in-vivo EC_90_ derived from data by Min and colleagues^19^ was approximately 0·32 mg/L.^19^ In addition, within a phase 2b dose-ranging trial, the GM C_trough_ in adults on the lowest dolutegravir dose tested (10 mg film-coated tablet once daily) was 0·30 mg/L, which resulted in sustained virological response rates similar to 50 mg film-coated tablet once daily at 96 weeks of treatment (79% *vs* 88%, with plasma HIV-1 RNA <50 copies per mL). No dose-related toxic effects were identified and the 50 mg dose was subsequently chosen because of robustness against missed doses and possible reductions in drug exposure caused by drug-drug interactions.^26^ With a conservative approach, considering 0·32 mg/L as minimum C_trough_ target, our results suggest that the paediatric dolutegravir doses approved at the time of the study can result in low C_trough_ in around a third of children on 25 mg or 35 mg film-coated tablets; by contrast, only one of 35 children had low C_trough_ on the adult 50 mg film-coated tablet.

The most likely explanation for children requiring relatively high doses to achieve similar trough concentrations compared with adults is their higher dolutegravir plasma clearance per kg of bodyweight.^27^ Of note, there is an increase in dolutegravir exposure when it is administered with food. In adult healthy volunteers, food increased dolutegravir C_24_ by 33% with low, 45% with moderate, and 73% with high fat meals compared with fasting.^28^ In the ODYSSEY trial, we studied pharmacokinetics under fasted conditions to compare with data on fasted adults and to represent the lowest likely scenario for dolutegravir trough concentrations. In real-life settings, dolutegravir concentrations might be higher in non-fasting children, although food security is an issue in many settings.

Important for interpretation of our data is the absence of a clear relationship so far between dolutegravir plasma concentration and adverse events or toxic effects.^22^, ^29^ Although the potential long-term toxicity of dolutegravir is still being explored, adults with virological resistance against first-generation integrase inhibitors receiving dolutegravir 50 mg twice daily for a median of 1 year had a reassuring safety profile, which was comparable to adults on once-daily dolutegravir.^20^ In our pharmacokinetic studies, children weighing 20 kg to less than 40 kg on increased doses of dolutegravir of 50 mg film-coated tablet and 30 mg dispersible tablets had GM AUC_0–24h_ in between reference values in adults on once-daily and twice-daily dolutegravir. Although GM C_max_ in children weighing 20 kg to less than 25 kg on 50 mg film-coated tablet and 30 mg dispersible tablets exceeded the reference parameters in adults on twice-daily dolutegravir, safety data are reassuring. Over a total follow-up of 20·5 patient-years in 47 children on the 50 mg film-coated tablet or 30 mg dispersible tablets none of the three reported adverse events were considered to be related to dolutegravir, and none were in the children weighing 20 kg to less than 25 kg. On the basis of results of these studies, and after review of data by the Independent Data Monitoring Committee and the Trial Steering Committee, children in the main ODYSSEY trial weighing 20 kg or more were switched to dolutegravir 50 mg film-coated tablet and safety and efficacy data collection is ongoing.

The use of the adult 50 mg film-coated tablet, which is currently the only formulation of single-entity dolutegravir available in LMICs, will greatly accelerate timely access to dolutegravir for children living with HIV.

Supplies for adults are less prone to stock-outs than paediatric formulations. The 50 mg dose aligns with adult dosing approved by the SRA of several ART backbone components for children; for instance with co-formulated tenofovir alafenamide 25 mg and emtricitabine 200 mg in children weighing 35 kg or more by the European Medicine Agency and in children weighing 25 kg or more by the FDA,^30^, ^31^ with abacavir 600 mg and lamivudine 300 mg for children weighing 25 kg or more,^32^ or tenofovir disoproxil fumarate 300 mg and lamivudine 300 mg for children weighing 35 kg or more. This dosing allows children to use adult single tablet regimens, such as abacavir 600 mg, lamivudine 300 mg, and dolutegravir 50 mg; tenofovir disoproxil fumarate 300 mg, lamivudine 300 mg, and dolutegravir 50 mg; as well as tenofovir alafenamide 25 mg, emtricitabine 200 mg, and dolutegravir 50 mg, which has recently received a tentative approval by the FDA.^33^ The latter two single-tablet regimens are produced by generic companies and expected to be widely available in low-resource settings.^5^ A single entity adult dolutegravir 50 mg film-coated tablet is already procured for adults requiring twice-daily dolutegravir for treatment of tuberculosis and integrase-resistant HIV, and therefore will be available for children weighing 20 kg or more.

Our data on the dispersible formulation of 30 mg in children weighing between 20 kg to less than 25 kg provide support that this dose is safe to be administered for all children weighing more than 20 kg who are unable to swallow film-coated tablets and add to the safety data on dolutegravir 50 mg film-coated tablets, because the 30 mg dispersible tablet dose and the 50 mg film-coated tablet have approximately equivalent bioavailability. Further pharmacokinetic studies evaluating dosing with dolutegravir 5 mg dispersible tablets have recently been completed in both IMPAACT P1093 and ODYSSEY and will be used to inform dosing in children weighing less than 20 kg. Scored dolutegravir 10 mg dispersible tablets are expected to be available from generic companies in 2020–21 providing an alternative to treatment based on ritonavir-boosted lopinavir for young children in LMICs. This should help to provide harmonised and practical treatment options for adults and children and optimal procurement for programmes in LMICs.

The results of the ODYSSEY pharmacokinetic and safety substudies in children weighing 20 kg to less than 40 kg were eagerly awaited by paediatric clinical communities, and have informed the recently updated WHO paediatric dosing recommendations.^13^ Based on the ODYSSEY data the FDA has recently approved the 50 mg film-coated tablet or 30 mg dispersible tablets for children weighing 20 kg or more.^34^ Further data are required on dolutegravir dosing in children with resistance to integrase inhibitors to evaluate whether the adult dose is safe and effective if given twice daily.

In conclusion, the adult dolutegravir 50 mg film-coated tablet given once daily provides adequate pharmacokinetic profiles in children weighing 20 kg or more with no safety signal of concern. Dolutegravir 30 mg dispersible tablets provide similar exposure to the 50 mg film-coated tablet and can be used in children unable to swallow tablets. The use of adult dolutegravir 50 mg film-coated tablets will allow practical dosing and rapid access to dolutegravir for children as well as alignment with adult ART regimens preferred by WHO in LMICs.

## Data sharing

The ODYSSEY data are held at Medical Research Council Clinical Trials Unit at University College London, which encourages optimal use of data by employing a controlled access approach to data sharing, incorporating a transparent and robust system to review requests and provide secure data access consistent with the relevant ethics committee approvals. All requests for data are considered and can be initiated by contacting mrcctu.ctuenquiries@ucl.ac.uk.
