# Supplementary material for: Simplified dolutegravir dosing for children with HIV weighing 20 kg or more: pharmacokinetic and safety substudies of the multicentre, randomised ODYSSEY trial
Source: Lancet HIV. 2020 Aug 4;7(8):e533–44. doi: 10.1016/S2352-3018(20)30189-2 (PMC7445428; doi:10.1016/S2352-3018(20)30189-2)
Supplement: Supplementary appendix [file mmc1.pdf]

THE LANCET
HIV

Supplementary appendix

This appendix formed part of the original submission and has been peer reviewed. We post it as supplied by the authors.

Supplement to: Bollen PDJ, Moore CL, Mujuru HA, et al. Simplified dolutegravir dosing for children with HIV weighing 20 kg or more: pharmacokinetic and safety substudies of the multicentre, randomised ODYSSEY trial. *Lancet HIV* 2020; **7:** e533–44.

Simplified dolutegravir dosing for HIV-infected children weighing ≥20kg; multicentre pharmacokinetic and safety substudies within the ODYSSEY randomised trial

Supplementary Appendix

CONTENT

1. Supplementary Table 1. Dose proportionality results for adult 50mg FCT *versus* 25mg and 35mg FCT
2. Supplementary Table 2: Equivalence testing results for adult 50mg FCT *versus* 30mg DT in children weighing 20 to <25kg
3. The ODDYSEY trial team

Supplementary Table 1. Dose proportionality results for adult 50mg FCT *versus* 25mg and 35mg FCT

	**20 to <40kg**
No. of children	**50**
Treatment comparison	50mg FCT *vs* 25/35mg FCT
No. included PK profiles	75[a]
No. within subject comparisons	25[a]
AUC_{0-24h} / Dose	0·91 (0·85-0·97)
C_{max} / Dose	0·89 (0·82-0·96)
C_{trough} / Dose	0·98 (0·87-1·10)
Dose proportionality[b]	Demonstrated

Data represent geometric mean ratios (GMR) with 90% confidence intervals for dose normalised PK parameters estimated with linear mixed models including dose group (50mg or 25/35mg) as a fixed-effect and a random effect for participant.
FCT, film-coated tablet.
a GMR (90% CI) for AUC_{0-24h} and C_{max} based on 24 within subject comparisons and 74 PK profiles.
b Dose proportionality is concluded if GMR (90%CI) for AUC_{0-24h} and C_{max} are both within the range of 0·80-1·25.

Supplementary Table 2. Equivalence testing results for adult 50mg FCT *versus* 30mg DT in children weighing 20 to <25kg.

	**20-<25kg**
No. of children	**18**
Treatment comparison	50mg FCT *vs* 30mg DT
No. included PK profiles	18
No. within subject comparisons	0
C_{trough} (mg/L)	0·99 (0·57-1·71)
AUC_{0-24h} (h*mg/L)	0·89 (0·70-1·15)
C_{max} (mg/L)	0·89 (0·68-1·15)
Equivalence[a]	Suggested

Data represent geometric mean ratios (GMR) with 90% confidence intervals for PK parameters estimated using independent t-test on log-transformed parameters.
FCT, film-coated tablet.
a Equivalence is concluded if GMR (90%CI) for AUC_{0-24h} and C_{max} is within the range of 0·80-1·25.

ODYSSEY TRIAL TEAM

The ODYSSEY Trial Team consists of: (MRC CTU) Diana M. Gibb, Deborah Ford, Abdel Babiker, Anna Turkova, Anna Parker, Helen Wilkes, Nasir Jamil, Clare Shakeshaft, Tasmin Phillips, Margaret Thomason, Samuel Montero, Joanna Calvert, Karen Scott, Joshua Gasa, Moira Spyer, Kaya Widuch, Reena Patel, Sarah Lensen, Shabinah Ali, Ben Wynne, Nadine Van Looy, Chiara Borg, Emma Little, Rebecca Turner, Cecilia L. Moore. (PENTA-ID) Carlo Giaquinto, Tiziana Grossele, Daniel Gomez-Pena, Davide Bilardi, Giulio Becchia. (INSERM-ANRS) Alexandra Compagnucci, Yacine Saidi, Yoann Riault, Alexandra Coelho, Laura Picault, Christelle Kouakam. (PHPT) Tim R. Cressey, Suwalai Chalermpantmetagul, Dujrudee Chinwong, Gonzague Jourdain, Rukchanok Peongjakta, Pra-ornsuda Sukrakanchana, Wasna Sirirungsi. (Sub-study Partners) Janet Seeley, Sarah Bernays, Magda Conway, Nigel Klein, Eleni Nastouli, Anita De Rossi, Maria Angeles Munoz Fernandez, David Burger, Pauline Bollen, Angela Colbers, Hylke Waalewijn. (Joint Clinical Research Centre, Uganda) Cissy M. Kityo, Victor Musiime, Elizabeth Kaudha, Annet Nanduudu, Emmanuel Mujyambere, Paul Ocitti Labeja, Charity Nankunda, Juliet Ategeka, Peter Erim, Collin Makanga, Esther Nambi, Abbas Lugemwa, Lorna Atwine, Edridah Keminyeto, Deogratiuos Tukwasibwe, Shafic Makumbi, Emily Ninsiima, Mercy Tukamushaba, Rogers Ankunda, Ian Natuhurira, Miriam Kasozi, Baker Rubinga. (Baylor College of Medicine Children's Foundation, Uganda) Adeodata R. Kekitiinwa, Pauline Amuge, Dickson Bbuye, Justine Nalubwama, Winnie Akobye, Muzamil Nsibuka Kisekka, Anthony Kirabira, Gloria Ninsiima, Sylvia Namanda, Gerald Agaba, Immaculate Nagawa, Annet Nalugo, Florence Namuli, Rose Kadhuba, Rachael Namuddu, Lameck Kiyimba, Angella Baita, Eunice Atim, Olivia Kobusingye, Clementine Namajja, Africanus Byaruhanga, Rogers Besigye, Herbert Murungi, Geoffrey Onen. (MUJHU Research Collaboration, Uganda) Philippa Musoke, Linda Barlow-Mosha, Grace Ahimbisibwe, Rose Namwanje, Monica Etima, Mark Ssenyonga, Robert Serunjogi, Hajira Kataike, Richard Isabirye, David Balamusani, Monica Nolan. (FAM-CRU, South Africa) Mark F. Cotton, Anita Janse van Rensburg, Marlize Smuts, Catherine Andrea, Sumaya Dadan Sonja Pieterse, Vinesh Jaeven, Candice Makola, George Fourie, Kurt Smith, Els Dobbels, Peter Zuidewind, Hesti Van Huyssteen, Mornay Isaacs, Georgina Nentsa,

Thabis Ncgaba, Candice MacDonald, Mandisa Mtshagi, Maria Bester, Wilma Orange, Ronelle Arendze, Mark Mulder, George Fourie. (PHRU, South Africa) Avy Violari, Nastassja Ramsagar, Afaaf Liberty, Ruth Mathiba, Lindiwe Maseko, Nakata Kekane, Busi Khumlo, Mirriam Khunene, Noshalaza Sbisi, Jackie Brown, Ryphina Madonsela, Nokuthula Mbadaliga, Zaakirah Essack, Reshma Lakha, Aasia Vadee, Derusha Frank, Nazim Akoojee, Maletsatsi Monametsi, Gladness Machache, Yolandie Fourie, Anusha Nanan-kanjee, Juan Erasmus, Angelous Mamiane, Tseleng Daniel, Fatima Mayat, Nomfundo Maduna, Patsy Baliram. (Prapokklao Hospital, Thailand) Chaiwat Ngampiyasakul, Pisut Greetanukroh, Wanna Chamjamrat, Praechadaporn Khannak. (Phayao Hospital, Thailand) Pornchai Techakunakorn, Thitiwat Thapwai, Patcharee Puangmalai, Ampai Maneekaew. (Chiangrai Prachanukroh Hospital, Thailand) Pradthana Ounchanum, Yupawan Thaweesombat, Areerat Kongponoi, Jutarat Thewsoongnoen. (Nakornping Hospital, Thailand) Suparat Kanjanavanit, Pacharaporn Yingyong, Thida Namwong, Rangwit Junkaew. (Khon Kaen Hospital, Thailand) Ussanee Srirompotong, Patamawadee Sudsaard, Siripun Nuanbuddee, Sookpanee Wimonklang. (Mahasarakam Hospital, Thailand) Sathaporn Na-Rajsima, Suchart_Thongpaen, Pattira Runarassamee, Watchara Meethaisong, Arttasid Udomvised. (Klerksdorp Tshepong Hospital Complex, South Africa) Ebrahim Variava, Modiehi Rakgokong, Dihedile Scheppers, Tumelo Moloantoa, Abdul Hamid Kaka, Tshepiso Masienyane, Akshmi Ori, Kgosimang Mmolawa, Pattamukkil Abraham. (Durban International Clinical Research Site, South Africa) Moherndran Archary, Rejoice Mosia, Sajeeda Mawlana, Rosie Mngqibisa, Rashina Nundlal, Elishka Singh, Penelope Madlala, Allemah Naidoo, Sphiwee Cebekhulu, Petronelle Casey, Collin Pillay, Subashinie Sidhoo, Minenhle Chikowore, Lungile Nyantsa, Melisha Nunkoo, Terence Nair, Enbavani Pillay, Sheleika Singh, Sheroma Rajkumar. (AHRI, South Africa) Osee Behuhuma, Olivier Koole, Kristien Bird, Nomzamo Buthelezi, Mumsy Mthethwa. (UZCRC, Zimbabwe) James Hakim, Hilda Mujuru, Kusum Nathoo, Mutsa Bwakura-Dangarembizi, Ennie Chidziva, Shepherd Mudzingwa, Themelihle Bafana, Colin Warambwa, Godfrey Musoro, Gloria Tinago, Shirley Mutsai, Columbus Moyo, Ruth Nhema, Misheck Nkalo Phiri, Stuart Chitongo, Joshua Choga, Joyline Bhiri, Wilber Ishemunyoro, Makhosonke Ndlovu. (HIVNAT, Thailand) Thanyawee Puthanakit, Naruporn

Kasipong, Sararut Chanthaburanun, Kesdao Nanthapisal, Thidarat Jupimai, Thornthun Noppakaorattanamanee, Torsak Bunupuradah, Wipaporn Natalie Songtaweesin, Chutima Saisaengjan. (European Site Investigators) Stephan Schultze-Straber, Christoph Konigs, Robin Kobbe, Felicia Mantkowski, Steve Welch, Jacqui Daglish, Laura Thrasyvoulou, Delane Singadia, Sophie Foxall, Judith Acero, Gosia Pasko-Szcech, Jacquie Flynn, Gareth Tudor-Williams, Farhana Abdulla, Srini Bandi, Jin Li, Sean O'Riordan, Dominique Barker, Richard Vowden, Colin Ball Eniola Nsirim, Kathleen McClughlin, India Garcia, Pablo Rojo Conejo, Cristina Epalza, Luis Prieto Tato, Maite Fernandez, Luis Escosa Garcia, Maria José Mellado Peña, Talia Sainz Costa, Claudia Fortuny Guasch, Antoni Noguera Julian, Carolina Estepa, Elena Bruno, Alba Murciano Cabeza, Maria Angeles Muñoz Fernandez, Paula Palau, Laura Marques, Carla Teixeira, Alexandre Fernandes, Rosita Nunes, Helena Nascimento, Andreia Padrao, Joana Tuna, Helena Ramos, Ana Constança Mendes, Helena Pinheiro, Ana Cristina Matos. (Local Site Monitors) Flavia Kyomuhendo, Sarah Nakalanzi, Cynthia Mukisa Williams, Ntombenhle Ngcobo, Deborah Pako, Jacky Crisp, Benedictor Dube, Precious Chandiwana, Winnie Gozhora. (Independent Trial Steering Committee Members) Ian Weller, Elaine Abrams, Tsitsi Apollo, Polly Clayden, Valériane Leroy. (Independent Data Monitoring Committee Members) Anton Pozniak, Jane Crawley, Rodolphe Thiébaut, Helen McIlleron. (Endpoint Review Committee Members) Alasdair Bamford, Hermione Lyall, Andrew Prendergast, Felicity Fitzgerald, Anna Goodman.
